# The Lack of Calcium Salts in the Food of Troops on Active Service as a Predisposing Cause of Enteric Fever and Dysentery

**Published:** 1911-02-04

**Authors:** G. Arbour Stephens

**Affiliations:** Hon. Physician Royal Cambrian Institution for the Deaf and Dumb, Major 3rd Welsh Field Ambulance, R.A.M.C.T.


### The Royal Army Medical Corps Section.

### THE LACK OF CALCIUM SALTS IN THE FOOD OF TROOPS ON ACTIVE SERVICE AS A PREDISPOSING CAUSE OF ENTERIC FEVER AND DYSENTERY.

By G. Arbour Stephens, M.D., B.S., B.Sc.(Lond.), Hon. Physician Royal Cambrian Institution for the Deaf and Dumb, Major 3rd Welsh Field Ambulance, R.A.M.C.T.

The subject of the food supply of troops on active service is naturally one of very great national importance, and any suggestion that may lead to some method of maintaining or improving the health of troops at the front is, I venture to think, worthy of some consideration. With that idea in mind I have thought it worth while to draw attention to the fact that in the food supplied to troops on active service there is a marked deficiency of calcium salts, a state of things that tends, in my opinion, to lower the health and disease-resisting power of the persons so supplied.

There is not much need at the present day of emphasising the important part played by the calcium salts in the metabolism of the human body. One has only to remember that the skeleton, the great and important framework of the body of which it forms such a large part, is mainly composed of calcium salts, and the metabolic processes that take place in that organ must be such as affect and are affected by these salts.

One finds these salts present also in the marrow which is so intimately associated, on the one hand, with the bones and, on the other hand, with the blood, and this latter, taking its origin as it does from such a lime-impregnated source, has been shown during recent years to depend on a good and proper supply of calcium salts for the carrying on of its work in a healthy manner.

The marked contrast between the composition of healthy and diseased bones is both interesting and instructive. In healthy bones analysis shows that calcium phosphate forms 57 per cent. of the whole structure, whereas in rickety bones it only forms 26 per cent. The chemical products resulting from any disease in a child who is suffering from rickets, where the lime salts are so markedly depleted, must necessarily be very different from those produced by the same disease in a healthy child, and it requires no very great effort of the imagination to realise that the injection of a powerful substance, such as antitoxin, must have a resultant effect which is quantitatively, and most probably qualitatively, different from that produced in a relatively healthy child. In this way can be explained the urticarial rash that sometimes occurs after an injection.

From fœtal life, when we find the early deposition of spicules, through that important and mysterious period of puberty when the growth of bones and body ceases, to the time of old age, when the bones become altered in shape as well as more brittle, the calcium salts are the great pivot round which all the others seem to work.

The first and second dentitions only tend to emphasise still further the value of the lime salts that are so necessary for the proper repair of bones by means of callus, for it has been shown that callus is very badly formed if the calcium contents of the blood are not up to standard. The value of lime has long been recognised by those who are interested in agricultural pursuits, and the farmer who neglects to lime his fields is certain to realise very quickly a financial, if not a physiological, deficiency, as evidenced by the quantity and quality of the milk. It is well known that fowls must have free access to lime if the owner is desirous of obtaining a good supply of eggs, and if it is so necessary to consider the calcium value of the food that is supplied to cows and fowls how much more necessary is it for us to consider the calcium value of the food that we ourselves partake of?

The food is the only means whereby the body is supplied with lime, and during infancy the only source is milk. The ash of milk has been shown on analysis to consist of 25 per cent. of lime salts, all of which is needed for the rapidly growing bones; but what of those infants whose lot it is in life to be filled with stuffs that have but very low feeding value? Among such infants it is no wonder that rickets is so rife, and even those children of the poor who are brought up on the breast are not much better off, for unless the mothers get their proper supply of lime-salted food the milk must necessarily be poor in quality.

From a national point of view this is of the utmost importance, for if the work turned out by poor machinery is bad, what can we expect of the workmanship of those who have been badly bred and badly fed?

Other foods that are of great lime-bearing value are eggs and cheese, in the ash analysis of which we find 30 and 20 per cent. of calcium respectively. Meat and cereals contain about 2 to 8 per cent., while the other foods have a still lower percentage of lime salts.

The food of the poor is conspicuous by the absence of these most nourishing foods, and as a consequence we find among them a high infantile death-rate, a large number of rickety children, a tendency to alcoholism, and, later in life, a large number of persons who suffer from ulcers of the leg.

Some time ago I drew attention in the *Westminster Review* to the character of the food of the majority of the people of India, and showed how that food was particularly deficient in lime-containing food; but I also tried to show how the national instinct of that people endeavoured to make up for that deficiency by indulging in the national habit of chewing or eating "pan," which consists of portions of the betel nut wrapped up in the leaves of the betel-piper plant with small pieces of lime.

But notwithstanding this habit—which is, on account of the poverty of the people, often inaccessible to many of them—we find a large number of ulcers of the legs, a fact that is markedly evidenced by their offensive smell.

Among that class of our own community which indulges in tea and bread and butter and such foods as do not contain lime salts we also find the leg ulcers very prevalent, and four years ago I was able to prove that the cure of these ulcers could be brought about by the internal administration of a salt of lime, viz. calcium iodide.

One point of importance in carrying out such a treatment is to realise that those who are very alcoholic do not respond very readily to the iodide, and in this connection it is worth while pointing out that, although alcohol interferes with the action of calcium salts, they, on the other hand, interfere with the destructive work of alcohol—in other words, the lime salts are a good antidote for alcohol. For health, therefore, it is necessary that the body be supplied with proper food, *i.e.* food that contains a sufficiently large amount of lime salts, and if the body does not get those salts it naturally cannot perform its functions properly, or, in other words, it becomes diseased.

That a deficiency of lime salts does predispose to disease is evidenced by the large amount of intestinal troubles that arises in rickety children, and the inflammatory conditions that follow a depletion of calcium salts by alcohol, *e.g.*, pneumonia. Our soldiers, drawn as they are in the majority of cases from the poorer classes, are no exception to the calcium law; and when, on active service, under conditions of great stress and strain they have to do their work on dietaries that are greatly deficient in calcium salts, it is no wonder that they fall a prey to intestinal troubles of an inflammatory nature, and so help to swell the number of enteric and dysenteric patients.

A mere glance at the dietary table of soldiers on active service is sufficient to enable one to realise the fact that the food supplied is sadly deficient in lime salts.

Breakfast consists of bread or biscuit and jam (very rarely cheese). Dinner consists of tinned, or sometimes fresh, meat with tinned vegetables. With the evening tea a biscuit is served out. Not one of the articles of diet mentioned in the table contains more than 5 to 7 per cent. of lime salts.

The drain of lime salts from a body badly supplied with such is increased by efforts of an exhaustive nature, and when to this drain is added the depletion that follows the drinking of alcohol it is no wonder that disease becomes rife. Even acid drinks, and especially vinegar, must be looked upon with great suspicion when drunk by an exhausted soldiery on the field.

What, then, can be done to prevent the recurrence of the ravages wrought by disease on the battlefield? Can anything be done to improve matters, if only to a small extent? Is the War Office prepared to consider suggestions that may have a good effect? Are the head officials prepared to rectify such errors of dietary as I have pointed out? These are questions that are entitled to an answer, for investigations during recent years have drawn attention to the necessity of calcium salts for the proper working of the human organism, and if that human organism in the form of a soldier is not given that which it is his right to have, a grave responsibility rests on the shoulders of those in authority. One cannot expect soldiers on active service to be supplied with fresh milk and eggs; but the lime salts in those foods can be substituted by a supply of tablets of calcium lactate which, together with a quantity of capsules of calcium permanganate, would materially diminish the amount of disease that occurs in war.

In order to test the value of the calcium lactate tablets I gave two to each member of a squad of twelve men who had entered for a marching competition, with instructions that one was to be taken fifteen minutes before the start and the other after half the distance had been covered. As a result the squad, although it did not come in first, came in freshest, and to be fresh and fit is after all the great essential.

In a district where the supply of water is polluted and the conditions of service such as to produce a great thirst, if the men were to take with each draught of water one or two capsules of calcium permanganate their chance of infection would be greatly diminished. When this drug, which is of great value in enteritis, is taken in capsules it does not present any difficulties in the form of an objectionable taste, such as occurs when chemicals are added to water on a wholesale scale, while there is the great advantage of allowing the drug to act in the fresh condition on that part that requires it most—namely, the intestinal mucous membrane.

In malarial districts the permanganate salt has an additional advantage in that it has been shown to be of great value in the treatment of that disease.

In conclusion, I would point out that the suggestions put forward in this paper have the great advantage of simplicity, together with that of economy, and as such are devoid of many of the objections associated with elaborate and expensive schemes.

